# Experimental Study on the Salt Freezing Durability of Multi-Walled Carbon Nanotube Ultra-High-Performance Concrete

**DOI:** 10.3390/ma15093188

**Published:** 2022-04-28

**Authors:** Guifeng Liu, Huadi Zhang, Jianpeng Liu, Shuqi Xu, Zhengfa Chen

**Affiliations:** 1 Department of Civil Engineering, Changzhou University, Changzhou 213164, China; lgfcczu@163.com (G.L.); zhd690660085@163.com (H.Z.); ljp@cczu.edu.cn (J.L.); 2 Anhui Jiaxiong Construction Engineering Co., Ltd., Hefei 230000, China; xsqahjx@163.com

**Keywords:** multi-walled carbon nanotubes, ultra-high-performance concrete, mechanical properties, salt erosion and freeze-thaw coupling, durability, microstructure

## Abstract

Ultra-high-performance concrete (UHPC) is a new type of high-performance cement-based composite. It is widely used in important buildings, bridges, national defense construction, etc. because of its excellent mechanical properties and durability. Freeze thaw and salt erosion damage are one of the main causes of concrete structure failure. The use of UHPC prepared with multi-walled carbon nanotubes (MWCNTs) is an effective method to enhance the durability of concrete structures in complex environments. In this work, the optimal mix proportion based on mechanical properties was obtained by changing the content of MWCNTs and water binder ratio to prepare MWCNTs UHPC. Then, based on the changes in the compressive strength, mass loss rate, and relative dynamic modulus of elasticity (RDME), the damage degree of concrete under different salt erosion during 1500 freeze-thaw (FT) cycles was analyzed. The changes in the micro pore structure were characterized by scanning electron microscope (SEM) and nuclear magnetic resonance (NMR). The test results showed that the optimum mix proportion at the water binder ratio was 0.19 and 0.1% MWCNTs. At this time, the compressive strength was 34.1% higher and the flexural strength was 13.6% higher than when the MWCNTs content was 0. After 1500 salt freezing cycles, the appearance and mass loss of MWCNTs-UHPC prepared according to the best ratio changed little, and the maximum mass loss was 3.18%. The higher the mass fraction of the erosion solution is, the lower the compressive strength and RDME of concrete after FT cycles. The SEM test showed that cracks appeared in the internal structure and gradually increased due to salt freezing damage. However, the microstructure of the concrete was still relatively dense after 1500 salt freezing cycles. The NMR test showed that the salt freezing cycle has a significant influence on the change in the small pores, and the larger the mass fraction of the erosion solution, the smaller the change in the proportion of pores. After 1500 salt freezing cycles, the samples did not fail, which shows that MWCNTs UHPC with a design service life of 150 years has good salt freezing resistance under the coupling effect of salt corrosion and the FT cycle.

## 1. Introduction

In areas with a cold climate and salinization, concrete materials are inevitably tested by salt erosion and freeze-thaw environments in various bridges, tunnels, hydraulic structures, and other projects. Freeze thaw and salt erosion damage are the main causes of concrete structure deterioration [1]. 

Scholars have investigated the durability of concrete under the action of a single factor or a combination of multiple adverse factors. Paul Brown et al. [2] studied concrete soaked in a sodium sulfate solution, and revealed change in the microstructure of concrete and the formation mechanism of erosion products. Gastaldini et al. [3] found that with the increase in freeze-thaw cycles, the chloride penetration resistance of concrete decreased in a freeze-thaw and chloride coupling environment. With an increase in the corrosion time, the chloride ion permeability of concrete also decreased gradually. Jan et al. [4] analyzed the mechanical properties of slag concrete in a salt freezing environment and found that the compressive strength of slag concrete gradually decreased under salt freezing. Sahmaran et al. [5] found that with an increase in the freeze-thaw cycles and corrosion time, the water cement ratio of concrete was small, and its durability was excellent. Ji et al. [6] studied the performance changes in carbon fiber-reinforced polymer (CFRP) in a chloride salt and freezing coupling environment. The chloride ion diffusion test showed that the chloride ion concentration of the ordinary specimen was at least 200 times higher than that of the fully strengthened specimen. 

However, research on the frost resistance durability of concrete under the coupling environment of freeze-thaw and erosion has mostly focused on ordinary concrete. The water binder ratio of high-performance cement-based materials (e.g., UHPC) is low compared with that of ordinary concrete, which is mainly attributed to the compact arrangement of particles [7]. Therefore, UHPC not only has good mechanical properties (compressive strength, flexural strength) but also has excellent durability [8]. However, UHPC is usually produced without coarse aggregate, so the price of UHPC is more expensive than ordinary concrete. Dong et al. [9] found that the initial construction cost of bridges constructed with UHPC was increased by about 80%. The life cycle cost of the two bridges was also studied. When the service life of these 2 bridges was less than about 120 years, the life cycle cost of UHPC was higher than that of conventional bridges. However, considering the equivalent annual cost, the cost of the UHPC bridge is less than that of an ordinary bridge because the UHPC bridge can prolong the service life of the structure. Therefore, UHPC is mostly used in aggressive environmental structures or important buildings that need to be highly durable [10]. 

In order to prepare cement-based materials with high strength and toughness, many researchers have focused on carbon nanomaterials. Carbon nanotubes (CNTs) are a carbon material discovered by the Japanese scientist Iijima in the 1990s [11]. According to the number of graphene layers, they are divided into single-walled carbon nanotubes (SWCNTs) and multi-walled carbon nanotubes (MWCNTs). Carbon nanotubes have excellent mechanical properties, with an elastic modulus of about 1tpa and a yield strain of 10%–20% [12,13,14]. Therefore, carbon nanotubes are an ideal composite-reinforcing material [15]. 

The research on carbon nanotube cement-based composites is still in the exploratory stage [16,17]. Scholars found that the mechanical properties of carbon nanotube cement-based composites are closely related to the content of carbon nanotubes. The flexural and compressive strength of the composite can be significantly improved when the content is about 0.08–0.2%, but the optimal content and reinforcement effect are not unified [18,19]. Morteza et al. [20] found that the optimum content of carbon nanotubes in cement matrix is about 0.2wt%, and the compressive strength is 23.7% higher than that of ordinary mortar samples. Wang et al. [21] found that the addition of treated carbon nanotubes significantly improved the fracture energy and flexural toughness index of cement paste. Guan et al. [22] found that when the content of carbon nanotubes was 0.05%, the early flexural and compressive strength increased by 70.7% and 25.6%, respectively. The addition of carbon nanotubes also improved the later flexural and compressive strength, with a maximum increase of 16.9% and 11.6%, respectively. The addition of carbon nanotubes also results in significant changes in the microstructure of concrete, which improves the mechanical properties and durability and prolongs the service life. Zhang et al. [23] found that the fiber bridging effect of carbon nanotubes makes the cement paste structure denser, reducing holes and cracks, and improving the toughness and strength of cement. Using the by mercury intrusion porosity method (MIP), Guan et al. [22] found that MWCNTs can reduce the total porosity and improve the pore size distribution, reducing the number of mesopores. In addition, crack bridging, filling, and bonding between MWCNTs and cement matrix were also found in an SEM test, which had a positive impact on the mechanical properties. Fakhim et al. [24] observed that MWCNTs functioned to bridge cracks in cement-based materials with broken fibers. When the content of MWCNTs is 0.1–0.3%, cement-based materials show the best impermeability. An excessive MWCNTs content will produce agglomeration and lead to large holes. Therefore, the use of carbon nanotubes to prepare UHPC has important research significance and social value to enhance the durability of concrete structures in complex environments.

In this paper, MWCNTs were used as fiber reinforcement to prepare UHPC, and the effects of its content and water binder ratio on the mechanical properties were studied. In addition, the time-varying deterioration law of the freeze-thaw durability of MWCNTs UHPC under the coupling effect of salt erosion and freeze-thaw is discussed. The microstructure damage mechanism was analyzed by field SEM and NMR.

## 2. Materials and Methods

### 2.1. Sample Preparation

**Figure 1:**
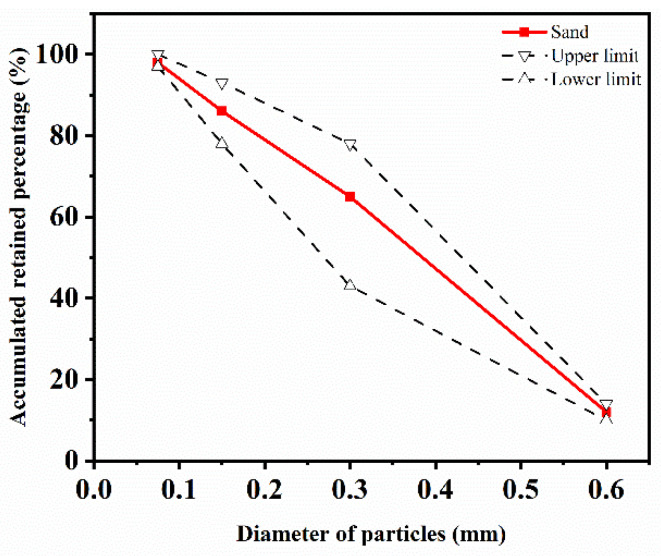
Granulometry curve of sand.

The carbon nanotube material was provided by a nano company in Shandong, and its physical parameters are shown in Table 1. Ordinary Portland cement P.O 52.5, complying with the Chinese standard GB175-2007 [25], was used in the experiment. Polyvinyl pyrrolidone (PVP) produced by Guangdong Co., Ltd. (Guangdong, China) was used as dispersant. A light-yellow viscous liquid polycarboxylic acid was used as a high-efficiency water-reducing agent, which was obtained from Qingdao Co., Ltd. (Qingdao, China). Local river sand 0.16–0.6 mm in size was used as fine aggregate. The granulometry curve is shown in Figure 1.

Based on the results of previous studies [27], carbon nanotubes were initially mixed with water, and then magnetically stirred with PVP of 30 °C for 15 min to promote surfactant dissolution, followed by ultrasonic treatment for 40 min. Finally, a carbon nanotube dispersion with different dosages was obtained. 

In total, 5 MWCNT UHPC with different water binder ratios were prepared, and MWCNTs was added at 0%, 0.02%, 0.05%, 0.10%, 0.15%, 0.20%, and 0.30% by cement weight. The mix proportions of all specimens are listed in Table 2. The materials were poured into 100 mm × 100 mm× 100 mm and 100 mm× 100 mm × 400 mm molds. All specimens were demolded after 24 h and then subjected to 28 days of standard curing (20 ± 2°C and 95% relative humidity).

Referring to the actual working conditions in the typical permafrost area of northeast China, a composite salt erosion solution containing sodium sulphate, sodium chloride, and sodium bicarbonate with a mass fraction of 3.4% [28] was prepared. In order to analyze the erosion damage degree and mechanism of various single salts of concrete, sodium bicarbonate, sodium chloride, and sodium sulphate solutions were prepared accordingly. Clear water solution was used as contrast. The composition and mass fraction of the five groups of erosion solution are shown in Table 3.

### 2.2. Test Apparatus

The main apparatus used in the test is shown in Table 4.

### 2.3. Experiment Program

After 28 days, the compressive strength and flexural strength of the specimen were tested with different water binder ratios and different contents according to the Chinese standard GB/T 50081-2019 [29]. The loading speed was 0.05–0.08 MPa/s.

Specimens with a size of 100 mm × 100 mm × 400 mm were prepared based on the optimal water binder ratio and the optimal content of MWCNTs. The samples were subjected to the rapid FT cycle test according to the Chinese standard GB/T 50082-2009 [30]. The temperature range of the fast FT test was −18 ± 2∼5 ± 2 °C. The ratio of one FT cycle test in the laboratory to the number of FT cycles in the natural environment is generally 10~15, and typically 12 [31]. The annual freezing and thawing times in northeast China is 120 combined with statistical data [32,33]. Therefore, the number of laboratory FT cycles is 1500 for structures with a design service life of 150 years. After every 500 FT cycles, the micro morphology was observed, and the compressive strength was tested. After every 50 FT cycles, the mass loss rate and RDME were tested until the FT cycle reached 1500 times or the test piece failed (mass loss ≥ 5% or RDEM ≤ 60%).

A diamond saw was used to prepare a specimen slice with a size of 8 mm × 8 mm × 5 mm. The sample slices were cleaned with an ultrasonic wave to prevent dust pollution affecting the test results. The slices were dried for 24 h and then sprayed gold. The micro morphology of MWCNTs UHPC in high vacuum mode was photographed by SEM.

After the FT cycle of the specimen, a 40 mm × 40 mm × 40 mm cube specimen was prepared using the cutting mechanism. A nuclear magnetic resonance imaging analyzer was used to scan the concrete samples. The resonance frequency was 12.00 MHz, the magnet temperature was 32.00 ± 0.01 °C, and the diameter of the probe coil was 25 mm. Vacuum pressure saturation was used to pressurize the concrete sample at 10 MPa and saturate it for 24 h. The pore size distribution of MWCNTs UHPC before and after the freeze-thaw was characterized by the NMR relaxation method. In the NMR test, the relaxation time T_2_ value is positively correlated with the pore size. The larger the peak value of the T_2_ spectrum, the larger the pore size in the sample. The greater the amplitude, the greater the number of pores in the material [34,35].

**Figure 2:**
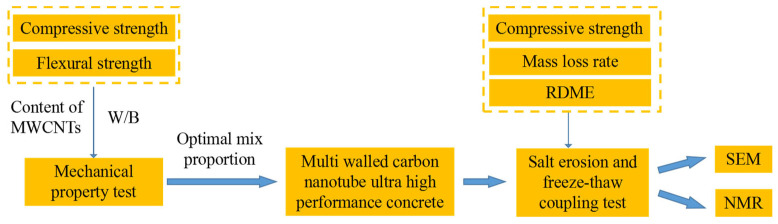
Test flow chart.

The test flow chart is shown in Figure 2.

## 3. Results and Discussion

### 3.1. Optimal Mix Proportion Based on Mechanical Properties in a Non-Salt Freezing Environment

**Figure 3:**
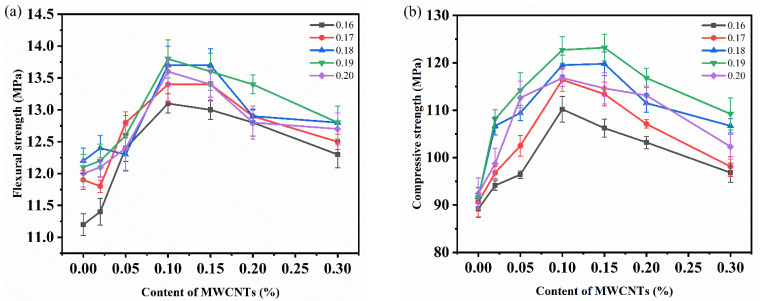
Mechanical properties of MWCNTs UHPC with different water binder ratios (0.16–0.20) and different MWCNTs contents (0–0.30%): (**a**) Flexural strength; (**b**) Compressive strength.

The compressive strength and flexural strength of the specimens with different water binder ratios and different contents without a salt freezing cycle were tested to investigate the effects of the water binder ratio and carbon nanotube content on the mechanical properties. Figure 3 shows that the addition of MWCNTs significantly improved the compressive strength and flexural strength of concrete. After increasing the carbon nanotubes content, the compressive strength and flexural strength increased at first and then decreased, which is consistent results from the literature [36,37,38]. The maximum compressive strength and flexural strength were 123.2 and 13.9 MPa, respectively, which was shown by the specimen with a water binder ratio of 0.19 and MWCNTs content of 0.1%. The strength was increased by 34.6% and 11.2%, respectively, compared with the control group with an MWCNTs content of 0. When the water binder ratio was 0.19 and the MWCNTs content was 0.1%, the compressive strength of the specimen reached the second highest value of 122.7 MPa and the flexural strength reached the highest value of 14.2 MPa. The strength was increased by 34.1% and 13.6%, respectively, compared with the control group.

This is because MWCNTs are evenly dispersed in concrete when the MWCNTs content is small (0–0.1%). Moreover, the uniformly dispersed MWCNTs fill the concrete interface transition zone, pores, and cracks, enhancing the mechanical properties. When the MWCNTs content is too high (0.1–0.3%), it is difficult for the MWCNTs suspension system to maintain stability and the MWCNTs can easily entangle and agglomerate with each other. As a result, there are a large number of non-dense and honeycomb pores and holes in cement hydration products. This decreases the interface compatibility between MWCNTs and concrete matrix, which is unfavorable regarding the mechanical properties. 

At the same time, it can be seen from the figure that the compressive strength and flexural strength of the specimen first increased and then decreased with the increase in the water binder ratio, and reached a peak value when the water binder ratio was 0.18 or 0.19. Generally, the water binder ratio of UHPC should be less than 0.2. However, the fluidity of the concrete mixture decreases, and the interface is incompatible as there is less water in the concrete mixture when the water binder ratio is very low (such as when the water binder ratio was 0.16 and 0.17). A large number of pores will form in the interior due to the non-compactness of the mixture during the preparation of concrete specimens, resulting in a reduction in the UHPC strength. However, the fluidity of UHPC increases when the water binder ratio increases. Cementitious materials and aggregates are closely connected, which greatly improves the homogeneity of UHPC. Therefore, the strength of concrete is improved. However, the amount of mixing water increases and the strength of concrete decreases when the water binder ratio increases further.

Similar conclusions have been made in the studies of other scholars. Ju et al. [39] stated that an increase in W/B in a certain range results in the hydration of cementitious materials being more complete, thereby increasing the content of C-S-H gel and AFt in cement hydration products. To increase the strength of the test piece, Lu et al. [40] found that the early compressive strength of UHPC is largest when the water binder ratio is 0.18 while the flexural strength is highest when the water binder ratio is 0.2. The is because when the water binder ratio is small, there is not enough movable free water in the system. With the increase of water binder ratio, the movable free water in the system increases.

Little difference was observed in the mechanical properties of the concrete specimens when the water binder ratio was 0.19 and the MWCNTs content was 0.1% and 0.15%. Therefore, considering the cost factor (when the strength difference is small, the MWCNTs content should be as small as possible), a water binder ratio of 0.19 and MWCNTs content of 0.1% were selected as the optimal mix proportion. Then, based on the optimal mix proportion of MWCNTs UHPC, the freeze-thaw durability of concrete in a salt erosion and freeze-thaw coupling environment was investigated.

### 3.2. Rapid FT Test

#### 3.2.1. Morphological and Compressive Strength

**Figure 4:**
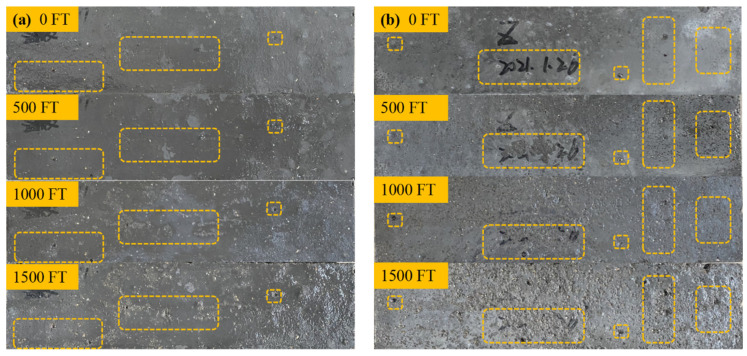

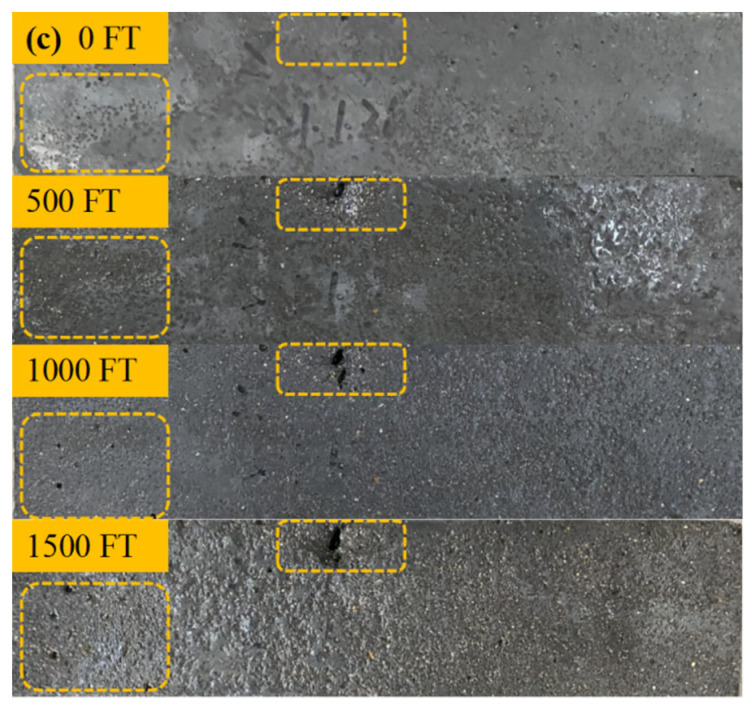
Specimen morphology of MWCNTs UHPC under different salt freezing cycles: (**a**) Bicarbonate and FT coupling; (**b**) Chloride salt and FT coupling; (**c**) Clean water and FT coupling.

Concrete specimens with different solutions and different FT cycles were photographed and recorded. The micro morphologies of specimens after 0, 500, 1000, and 1500 freeze-thaw cycles in a salt freezing-thawing environment were compared and analyzed. The morphological changes are shown in Figure 4. The dotted box shows the morphological changes of some obvious pores after different salt freezing cycles. It can be seen that the appearance of the concrete specimens did not change significantly during the FT cycle. After zero salt freezing cycles, the surface of the specimen was smooth, and only small pores were generated in the process of preparation and curing. With an increase in the salt freezing cycles, small pores on the surface of the specimen gradually developed into medium pores and large pores. Moreover, the number of small pores increased gradually. The mortar was gradually exposed, and the surface became uneven due to salt freezing damage. However, no large cracks appeared on the surface of the specimen and the integrity of the specimen was not damaged after 1500 salt freezing cycles, indicating that MWCNTs-UHPC has good salt freezing resistance.

**Figure 5:**
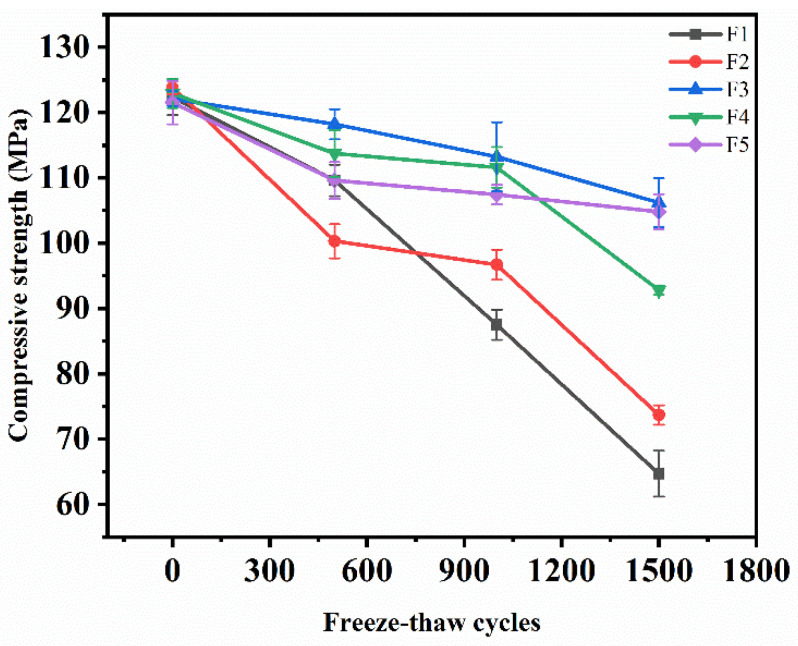
Variation in the compressive strength during salt freezing cycles. (F1: Composite salt and FT coupling; F2: Bicarbonate and FT coupling F3: Chloride salt and FT coupling; F4: Sulphate and FT coupling; F5: Clean water and FT coupling).

Compressive strength is an important index that measures the bearing capacity of concrete. The change in the compressive strength with the number of salt freezing cycles is shown in Figure 5. It can be seen from the figure that the compressive strength of concrete decreased with the increase in the salt freezing cycles. The loss rates of compressive strength were 47%, 40%, 13%, 24%, and 14%, respectively. The variation range of the compressive strength from large to small is: composite salt and FT coupling > bicarbonate and FT coupling > sulphate and FT coupling > chloride and FT coupling > clean water and FT coupling. This shows that the greater the mass fraction of the erosion solution, the greater the loss of compressive strength.

#### 3.2.2. Mass Loss Rate

**Figure 6:**
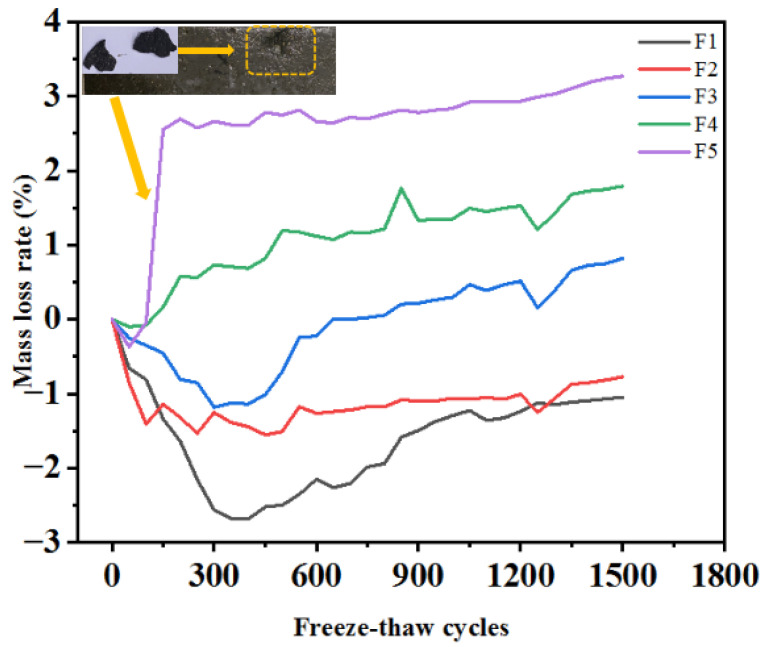
The mass loss rate of MWCNTs UHPC during salt freezing cycles. (F1: Composite salt and FT coupling; F2: Bicarbonate and FT coupling F3: Chloride salt and FT coupling; F4: Sulphate and FT coupling; F5: Clean water and FT coupling).

Quality change is an important indicator of concrete deterioration in salt frozen areas [41]. The peeling of mortar on the concrete surface caused by the FT effect is the main reason for a change in the quality [42]. The mass change trend of the test pieces under different solution FT cycles is shown in Figure 6. With the salt freezing cycles, the mass loss rate of all specimens decreased first and then increased. After 1500 FT cycles, the mass loss rates of the specimens were −1.09%, −0.85%, 0.73%, 1.73%, and 3.18%, respectively. The curve decreased rapidly in the early stage of erosion and the range of change was small in the later stage. This is because in the early stage of the FT cycle, cracks appeared and expanded on the interior and surface of the specimen, so water continued to penetrate the interior of the specimen. Salt solution crystallization attached to the surface of the specimen. In addition, the mortar on the surface of the specimen peeled less, and the peeling quality was much lower than that of the water penetrating inside the specimen. Therefore, the mass of the specimen increased, indicating that the mass loss rate was negative, and continuously decreased. As shown in Figure 5, the mortar of the F5 specimen peeled off, resulting in its quality degradation after 150 salt freezing cycles. However, under the subsequent FT cycle, the change in the mass loss rate was insignificant. In the later stage of the FT cycle, the mortar continued to peel off due to the effect of salt freezing damage. The mass of the specimen decreased gradually, which shows that the mass loss rate increased. Nevertheless, the mass loss rate of all specimens did not reach 5% after 1500 salt freezing cycles. This indicates that all specimens were unbroken according to GB/T 50082-2009 [30], and no specimen failed after 1500 salt freezing cycles. The results demonstrated that MWCNTs-UHPC has good resistance to salt freezing erosion under long-term salt freezing.

Compared with the compressive strength loss rate and mass loss rate of UHPC after FT cycles reported by Lu et al. [43,44,45,46], as shown in Table 5, it was found that the mass loss rate of MWCNTs-UHPC in the composite salt freezing-thawing environment in this experiment was lower. In addition, a greater loss in compressive strength was found in this experiment. This is because the coupling effect of salt corrosion and freeze-thaw causes more serious damage to the concrete compared with the single freeze-thaw environment and reduces the strength.

#### 3.2.3. Relative Dynamic Modulus of Elasticity

**Figure 7:**
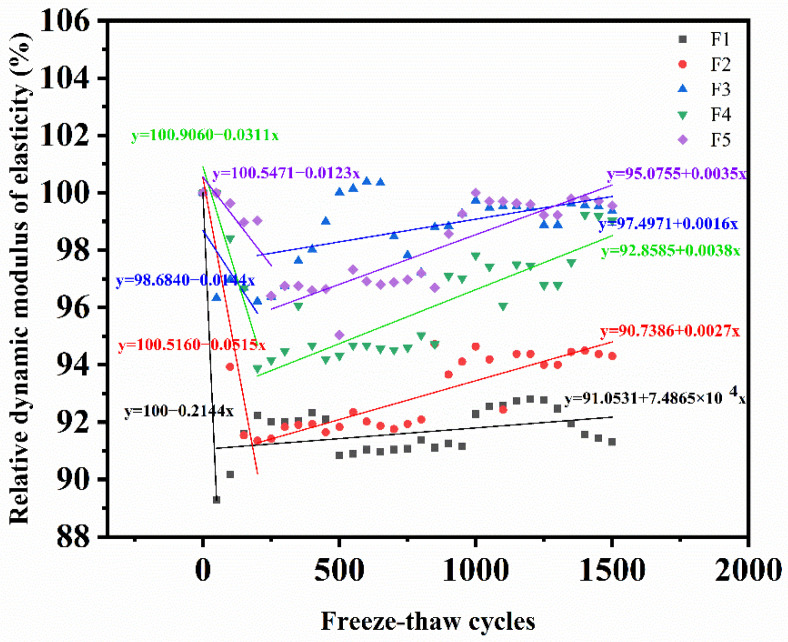
RDME of MWCNTs UHPC during salt freezing cycles (F1: Composite salt and FT coupling; F2: Bicarbonate and FT coupling F3: Chloride salt and FT coupling; F4: Sulphate and FT coupling; F5: Clean water and FT coupling).

When the size and quality are certain (no serious peeling occurs), RDME is only affected by the compactness of concrete. In order to better reflect the internal compactness and damage of MWCNTs UHPC specimens after salt freezing cycles, RDME under different solution freezing and thawing cycles was tested. The test results are shown in Figure 7. 

RDEM of the specimen decreased obviously in the early stage of the salt freezing cycles, and then increased slightly. After 1500 salt freezing cycles, RDME of the composite salt invasion and single salt invasion specimens was 91.3%, 94.3%, 99.36%, 99.03%, and 99.55%, respectively. The larger the mass fraction of the salt solution, the smaller the slope value of the downward trend line and the more obvious the downward trend. The minimum slope of the F1 compound salt solution is −0.2144 while the maximum slope of the F5 clear water solution is −0.0123. In the rising stage, the larger the mass fraction of the salt solution, the smaller the slope of the rising trend line. The minimum slope of the F1 compound salt solution is 7.4865 × 10^−4^ while the slope value of the F5 clear water solution is 0.0035. This is because the salt freezing damage of the concrete is related to the type of erosion salt, the mass fraction of the erosion solution, and other factors. The more complex the salt composition, the greater the mass fraction of the erosion solution, and the more serious the frost heave damage to the concrete. This rule is consistent with the change in the compressive strength.

The rising process of RDEM may be due to secondary hydration [47]. A large number of cementitious materials were not fully hydrated during the test because of the low water cement ratio of UHPC [48]. With the increase in the salt freezing cycles, the frost heaving force of water increases. The internal porosity of concrete increases and absorbs more water during salt freezing cycles, which promotes the hydration reaction of cementitious materials. The continuous hydration of the specimen matrix gradually increases the compactness of the specimen, which plays a role in improving the relative dynamic elastic modulus. It shows that MWCNTs UHPC has good frost resistance and salt erosion resistance under the combined action of composite salt and low temperatures.

### 3.3. Microscopic Analysis

#### 3.3.1. Scanning Electron Microscope Observation

**Figure 8:**
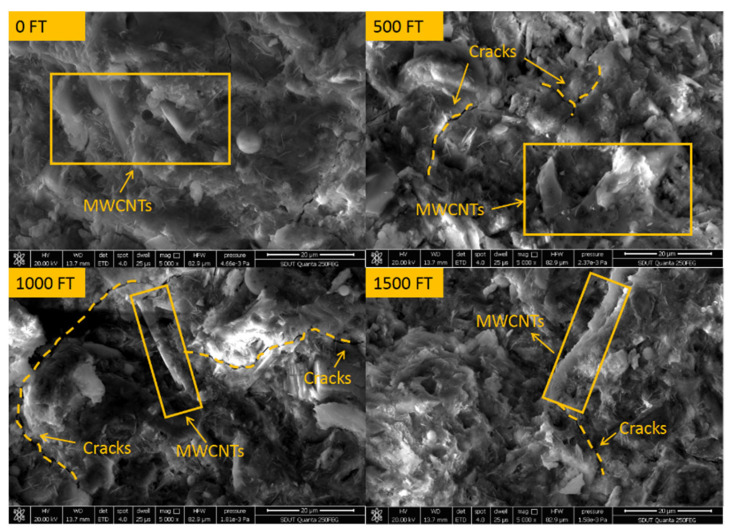
SEM of MWCNTs UHPC under different salt freezing cycles.

Figure 8 shows the microstructure of concrete under different salt freezing cycles. It can be seen from the figure that MWCNTs mostly overlap and form bridges with hydration products in the form of a single root to form multiphase composites and improve the mechanical properties of concrete [49]. Before the salt freezing cycles, the microstructure of concrete is dense without obvious pores and cracks. With the increase in the salt freezing cycles, cracks appear in the internal structure and further expand, and the connection between hydration products becomes loose and honeycomb. However, after 1500 salt freezing cycles, the hydration products are still relatively dense, and there is no looseness or porosity. This shows that for MWCNTs UHPC, due to the compactness of its structure, the diffusion rate of the erosion medium is reduced, and then the deterioration process of concrete FT damage is prolonged.

#### 3.3.2. Nuclear Magnetic Resonance Test

**Figure 9:**
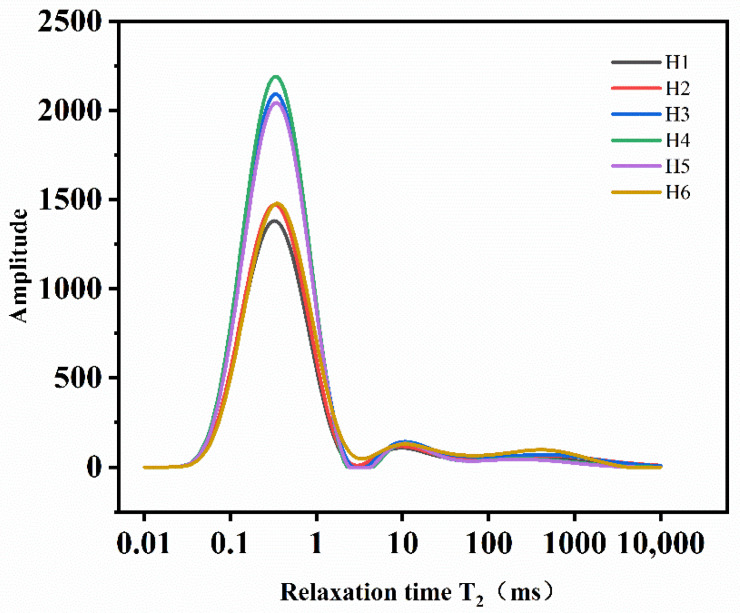
T_2_ distribution curve of the NMR test (H1-H5 is the sample after 1500 salt freezing cycles under 5 erosion solutions, and H6 is the control sample without salt freezing cycles).

Figure 9 shows the T_2_ distribution curve of the MWCNTs UHPC samples. As can be seen from the figure, there are three peaks in the T_2_ distribution diagram, in which the left peak represents the distribution of small holes, the middle peak represents the medium pores, and the right peak represents the large pores. As can be seen from the figure, the middle peak and right peak change little. This shows that salt freezing has little effect on these two peaks, so it is ignored. After 1500 salt freezing cycles, the number of small pores in the concrete samples in the single salt freezing environment increased while the number of small pores in the composite salt freezing environment decreased. This may be because in the composite salt erosion environment, corrosion crystals are more likely to be generated inside the sample and fill in small pores, thus reducing the number of small pores.

**Figure 10:**
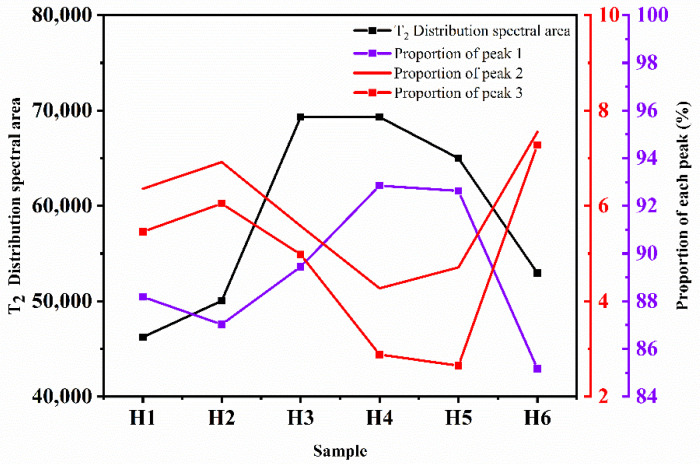
T_2_ spectrum area and proportion of the peak value of the T_2_ distribution.

In order to reflect the proportion change in the pores with different sizes, the peak data were analyzed. The analysis results are shown in Figure 10. As can be seen from Figure 10, the proportion of medium pores and macro pores in the sample after the FT cycle is slightly reduced while the proportion of small pores is increased compared with the control sample without the FT cycle. This is because the concrete will be continuously damaged with the development of the FT cycles, and more micro pores will form. Therefore, the proportion of small holes will increase.

In the composite salt, bicarbonate erosion, and FT coupling environment, the area of the T_2_ distribution spectrum decreases and the range of change in the pore ratio is small. In the chloride, sulfate, and clean water erosion and FT coupling environment, the area of the T_2_ distribution spectrum increases and the pore ratio changes significantly. This is because the mass fraction of the salt solution is large, and the salt composition is complex in the composite salt, bicarbonate erosion, and freeze-thaw coupling environment. With the salt freezing cycle, the ions in the salt solution enter the concrete and react chemically with the internal materials. The internal pores are constantly filled by corrosion crystals, which causes frost heave damage. At the same time, the macro pores gradually transform into small pores due to the accumulation of crystals. Therefore, the range of change in the pore ratio is small. The mass fraction of the salt solution is relatively small in the chloride, sulfate, clean water erosion, and freeze-thaw coupling environment. Although some crystals fill in the pores, the frost heaving damage to the concrete is more serious, and small holes are constantly generated with the progression of the FT cycle. Therefore, the proportion of small holes changes significantly.

## 4. Conclusions

In this study, the mechanical properties and frost resistance durability of UHPC containing MWCNTs were studied, and the micro analysis was carried out combined with SEM and NMR. The conclusions are as follows:

1. The addition of carbon nanotubes significantly improved the compressive strength and flexural strength of concrete. With the increase in the MWCNTs content, the variation curves of the compressive strength and flexural strength of the specimens increased at first and then decreased. Considering the cost factor, the optimum mix proportion was found to be a 0.19 water binder ratio and 0.1% carbon nanotube content. At this time, the compressive strength of the specimen was 122.7 MPa and the flexural strength was 9.2 MPa. The strength was increased by 34.1% and 13.6%, respectively, compared with the control group with an MWCNTs content of 0.
2. The MWCNTs UHPC prepared based on the optimal mix proportion showed good frost resistance and salt erosion resistance under the combined action of salt erosion and low temperatures. After 1500 salt freezing cycles, the appearance and mass loss of concrete did not change, and the maximum quality loss was 3.18%. The more complex the salt composition and the greater the mass fraction of the erosion solution, the higher the loss rate of compressive strength, up to 40%. This reduces RDME to 91.3%.
3. After 1500 salt freezing cycles, the microstructure of concrete was still dense. The salt freezing cycle has a significant influence on the change in the small pores but has little influence on the change in the medium pores and large pores. The larger the mass fraction of the erosion solution, the smaller the change in the pore proportion.

## Figures and Tables

**Table 1:** Physical parameters of MWCNTs [26].

Pipe Diameter /nm	Tube Length /µm	Purity /%	Ash /%	Specific Surface Area /m^2^·g^−1^	Packing Density /g·cm^−3^
10–20	5–50	>85	<2.0	200–300	0.006–0.09

**Table 2:** Mix proportions of the specimens (kg/m^3^).

Sample	Cement	Silica Fume	Fly Ash	Slag Powder	Sand	W/B	Water Reducer	MWCNTs Content (%)
M1	540	100	180	100	1080	0.16	2.5%	0/0.02/0.05/0.10/0.15/0.20/0.30
M2	540	100	180	100	1080	0.17	2.5%	0/0.02/0.05/0.10/0.15/0.20/0.30
M3	540	100	180	100	1080	0.18	2.5%	0/0.02/0.05/0.10/0.15/0.20/0.30
M4	540	100	180	100	1080	0.19	2.5%	0/0.02/0.05/0.10/0.15/0.20/0.30
M5	540	100	180	100	1080	0.20	2.5%	0/0.02/0.05/0.10/0.15/0.20/0.30

**Table 3:** Composition and mass fraction of erosion solution.

Sample	Erosion Solution Type	Type and Dosage of Salt /g·L^−1^			Solution Mass Fraction/%
		NaHCO_3_	NaCl	Na_2_SO_4_	
F1	Composite salt	14.38	7.46	13.36	3.4
F2	Bicarbonate	14.38	0	0	1.42
F3	Chloride salt	0	7.46	0	0.74
F4	Sulphate	0	0	13.36	1.32
F5	Clean water	0	0	0	0

**Table 4:** Main test apparatus.

Name	Model Parameters
Collector type constant temperature heating magnetic stirrer (Shanghai Yuhua Instrument Co., Ltd, Shanghai, China)	DF-101S
Ultrasonic cleaner (Shenzhen yuanpin Instrument Co., Ltd, Shenzhen, China)	KQ-250B
Single horizontal shaft forced concrete mixer (Wuxi Jianyi Instrument Machinery Co., Ltd, Wuxi, China)	HJW-60
Microcomputer controlled electro-hydraulic pressure testing machine (Shanghai Linjia science and Education Instrument Co., Ltd, Shanghai, China)	TYW-2000
Constant loading pressure testing machine (Wuxi xinluda Instrument Equipment Co., Ltd, Wuxi, China)	EHDC
Concrete freeze-thaw testing machine (Shanghai Sanhao refrigeration equipment factory, Shanghai, China)	CDR-5
Dynamic elastic modulus tester (Tianjin Yaxing Automation Experimental Instrument Co., Ltd, Tianjin, China)	DT-20W
Electron scanning microscope (Shanghai Baihe Instrument Technology Co., Ltd, Shanghai, China)	JSM-IT100(L)
Nuclear magnetic resonance imaging analyzer (Suzhou niumag Analytical Instrument Co., Ltd, Suzhou, China)	MesoMR12-060H-I

**Table 5:** The referred compressive strength loss rate and mass loss rate of UHPC after FT cycles.

Number	Author	Number of FT Cycles	Compressive Strength Loss Rate (%)	Mass Loss Rate (%)
1	Lu et al. [43]	300	27.5	−0.65
2	Lee et al. [44]	300	−3	-
		600	1	-
		1000	6	-
3	Ji et al. [45]	500	4.9–17.8	0.57–0.95
4	Li et al. [46]	800	0.869–1.501	-

## Data Availability

The data used to support the findings of this study are available from the corresponding author upon request.
